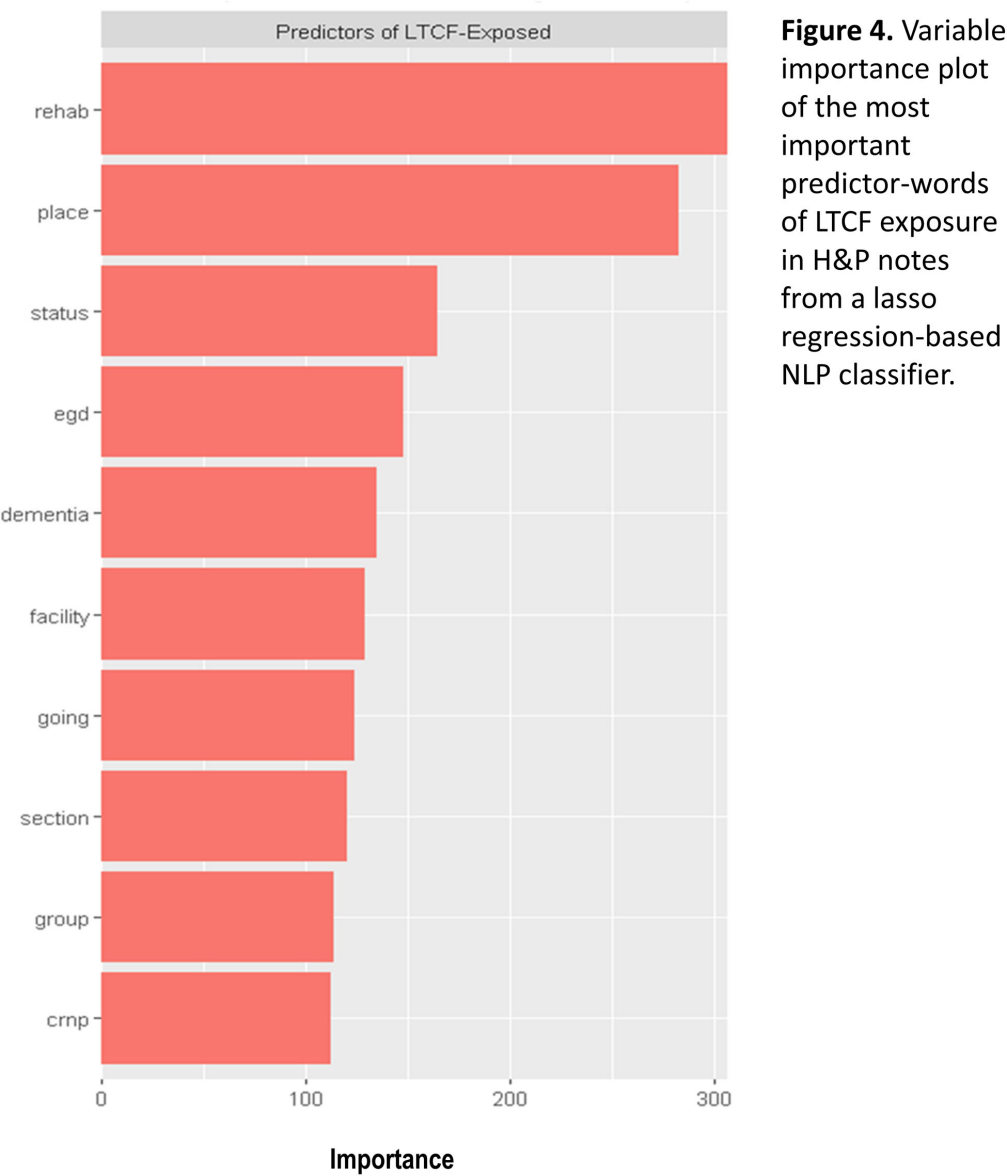# Natural Language Processing (NLP) Accurately Identifies LTCF Exposure from Clinical Notes: A Proof-of-Principle Study

**DOI:** 10.1017/ash.2024.114

**Published:** 2024-09-16

**Authors:** Katherine Goodman, Philip Resnik, Monica Taneja, Laurence Magder, Mark Sutherland, Scott Sorongon, Eili Klein, Pranita Tamma, Anthony Harris

**Affiliations:** University of Maryland School of Medicine; Johns Hopkins School of Medicine; Johns Hopkins

## Abstract

**Background:** Residence or recent stay in a long-term care facility (LTCF) is one of the most important risk factors for multidrug-resistant organism (MDRO) carriage and infection, making reliable identification of LTCF-exposed inpatients a critical priority for infection control day-to-day practice and research. However, because most hospital electronic health records (EHRs) do not include a dedicated field for documenting LTCF exposure, absent manual review of patient charts, identifying LTCF-exposed inpatients is challenging. We aimed to develop an automated, natural language processing (NLP)-based classifier for identifying LTCF exposure from clinical notes. **Methods:** We randomly sampled 1020 adult admissions from 2016-2021 across the 12-hospital University of Maryland Medical System and manually reviewed each admission’s history & physical (H&P) note for mention of LTCF exposure (Figure 1). After H&P pre-processing, we calculated feature representations for documents based on term frequencies and visually explored between-group (LTCF-exposed vs. LTCF-unexposed) feature differences. To predict LTCF status from the H&P notes, we trained and tuned a LASSO regression-based classifier on 70% of the data with 3-fold cross-validation and 1:1 up-sampling to address class imbalance. The final classifier was evaluated on the 30% held-out sample (not up-sampled), with calculation of the C-statistic (area-under-the-curve, AUC) with bootstrapped 95% confidence intervals, and construction of receiver-operating-characteristic and variable importance plots (R Version 4.3.2). **Results:** 7% (n=76 cases) of H&P notes documented LTCF exposure. In our visual analysis, the H&P words and phrases that were over-represented among LTCF patients had high face validity (Figure 2). The final LASSO-regression-based classifier achieved a C-statistic of 0.89 (95% CI: 0.80–0.98) on the held-out data for identifying LTCF exposure from the H&P notes (Figure 3). The most important model predictors (i.e., words) for distinguishing LTCF-exposed from LTCF-unexposed patients are reflected in Figure 4. The most important predictor-words of LTCF-exposure were “rehab,” “place,” “status,” “egd,” and “dementia.” **Conclusion:** In this multi-center study, even a simple NLP classifier demonstrated very strong discrimination for identifying LTCF exposure status from H&P notes, which could substantially reduce the manual review time required to identify LTCF-exposed inpatients. If automated in the electronic health record, it could also inform real-time MDRO screening decisions. Future research is planned to build more sophisticated classifiers using machine learning best practices, to build classifiers for additional MDRO risk factors, and to externally validate NLP classifiers on notes from an external healthcare system.

**Figure:**
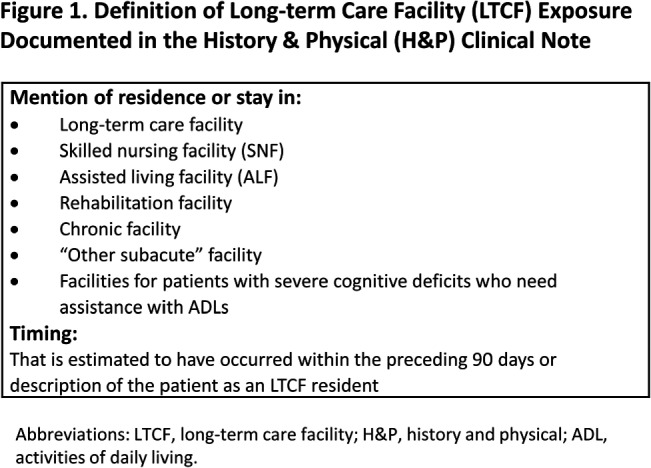

**Figure:**
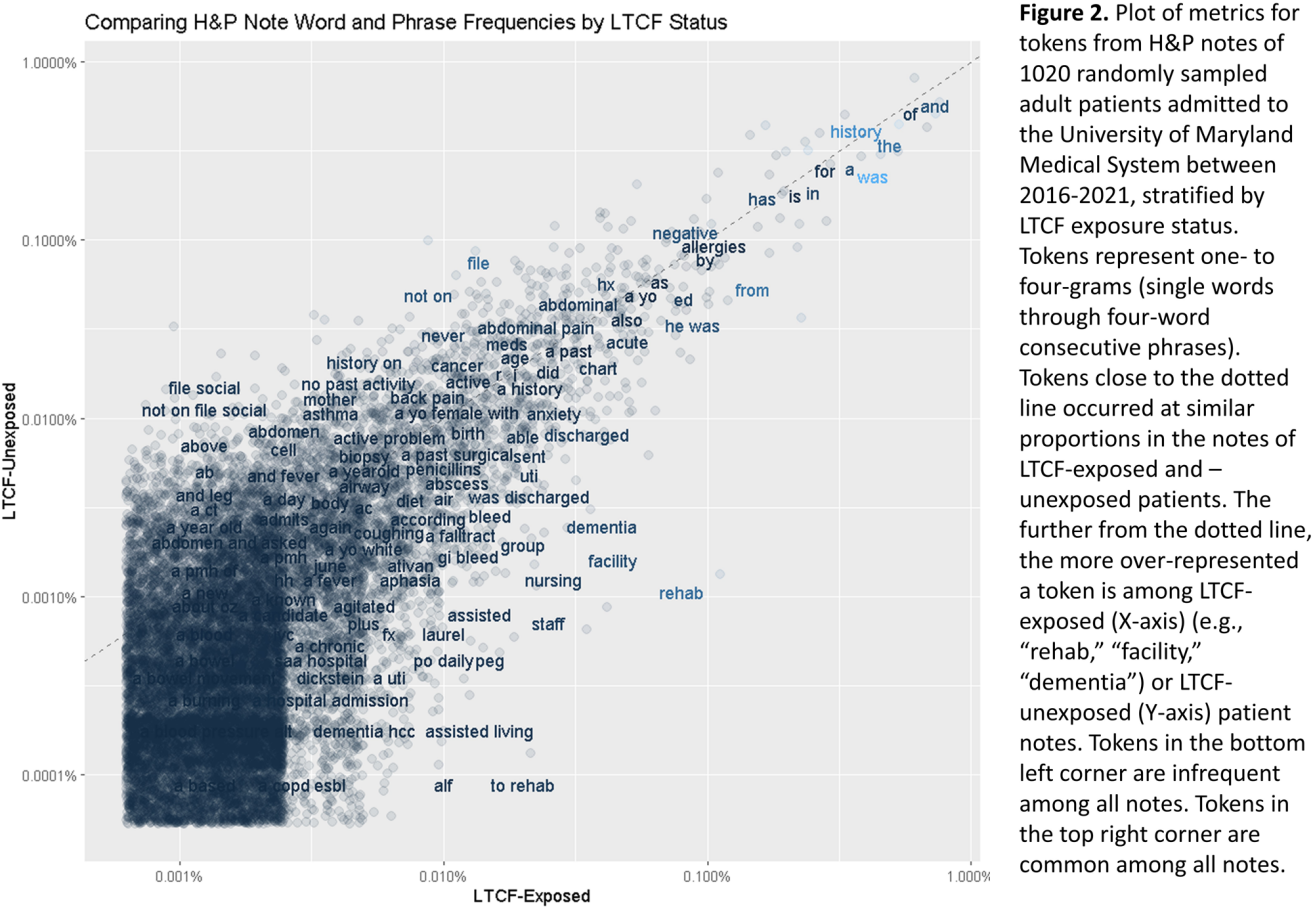

**Figure:**
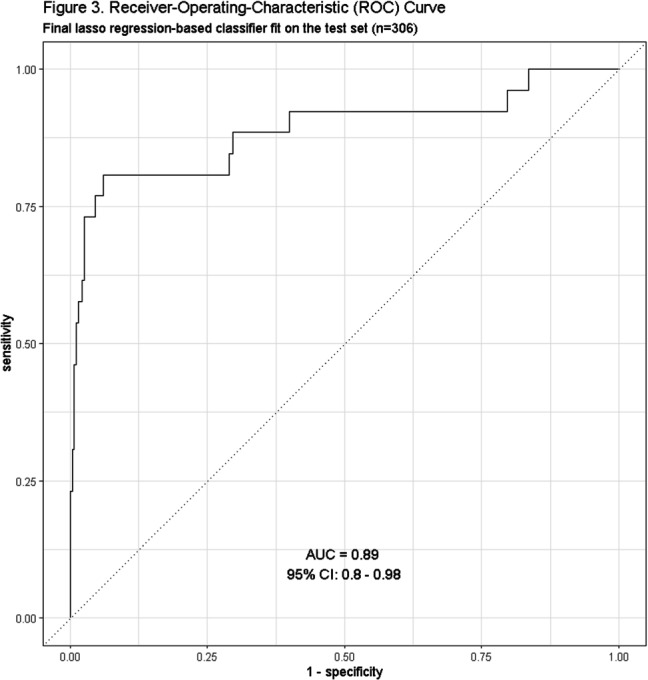

**Figure:**